# Diseases of the Ear

**Published:** 1857-11

**Authors:** 


ART. VII.—*Diseases of the Ear, illustrated by Clinical Observations.* By JOHN NOTTINGHAM, Surgeon to the Southern Hospital; Consulting Surgeon to the Eye and Ear Institutions, Liverpool. London: John Churchill, Burlington Street. 8vo. 644 pp. 1857.

"A book's a book although there's nothing in it."

WE are forcibly reminded of the truth of this couplet, by the perusal of the work of Mr. John Nottingham on the Diseases of the Ear. It has not fallen to our lot for a long time to peruse so large a work with so little substance, one so utterly jejune, and so entirely unsatisfactory in every particular. It is difficult to say whether the author intended it as a work for the benefit of his professional brethren, or as an advertisement for the acquisition of business. We are more than half inclined to believe that he was influenced by the latter consideration. If he had entitled his book a Collection of Cases of Diseases of the Ear, it would have conveyed to the reader a much better idea of the real character of the work, for it is really essentially this, and nothing more. The reader looks in vain for anything like a connected treatise on the subject, comprising a digest of the existing state of the science, or even a digest of the author's own experience. Why

Mr. Nottingham should have considered himself obliged to publish such a work, is beyond our power to conceive. We can hardly excuse a man for writing a book on any subject, unless he can offer something new and valuable, or present what is already known in a novel and interesting light. We cannot perceive that Mr. Nottingham has done either; the whole work is a continual repetition of cases, without any deduction of principles calculated to serve as a guide to sound aural medicine and surgery. The cases which he details are certainly not devoid of interest, but we defy any one, unless he is already familiar with the subject, to derive any material benefit from their study. Had he given us a comprehensive description of the various maladies of the ear, with well-selected clinical observations, he might have produced a work which would have compared favorably with that of Mr. Wilde, of Dublin, but, as it is, it is not to be named in the same year, much less in the same day. We pity Mr. Churchill, if he published the work at his own expense, for we are confident that the edition must be an entire failure, and we are equally certain that the work will never be reprinted on this side of the Atlantic, so distinguished for the liberal use it makes of the labors of our English brethren. We would advise Mr. Nottingham to remodel his book, and to give it at least something of the character which its present title would seem to imply. As it now stands, all that is really useful in it might be comprised in less than one-sixth of the space it occupies.

